# The effect of physical activity and motivation on function in ankylosing spondylitis: A cohort study

**DOI:** 10.1016/j.semarthrit.2012.09.007

**Published:** 2013-06

**Authors:** Sinead Brophy, Roxanne Cooksey, Helen Davies, Michael S. Dennis, Shang-Ming Zhou, Stefan Siebert

**Affiliations:** College of Medicine, Swansea University, Wales, UK

**Keywords:** Ankylosing spondylitis, Function, Motivation, Activity

## Abstract

**Objectives:**

Exercise is reported to improve function for people with ankylosing spondylitis (AS) but it is not clear if this effect is causal or if patients with milder disease find it easier to exercise. This study examines the effect of exercise and motivation to exercise on function, while controlling for disease severity.

**Methods:**

Participants who were members of an existing AS cohort were asked about physical activity, motivation to exercise, function, and disease severity. Path analysis on STATA was used to examine the correlation between factors associated with function at time of exercise and with function after 3 months of follow-up.

**Results:**

The response rate to the questionnaire was 88% (326/371). Improvement in function was greatest for people with higher physical activity levels and those who were more motivated to exercise—this was especially the case for patients with the most severe disease activity. The effect of motivation to exercise not only had a direct effect on function, but also an indirect effect of improving activity levels thereby improving both current and future function. People with high intrinsic motivation (driven by pleasure) had the greatest benefit to activity and function.

**Conclusions:**

Exercise does improve function, especially for those with severe disease. In addition, motivation alone improves function as much as exercising itself. Therefore, interventions targeting motivation to exercise would have as much effect on improving function as interventions offering exercise opportunities. In addition, any intervention that both improves motivation and increases opportunities to exercise would have a 2-fold influence on function.

Ankylosing spondylitis (AS) is the second most common inflammatory rheumatic disease and it is characterized by inflammatory back pain and sacroiliitis. It is associated with peripheral arthritis, enthesitis, uveitis, inflammatory bowel disease, and psoriasis [1].

Previous work has documented the beneficial effects of exercise on various aspects of health for AS patients, including physical function, disease activity, spinal mobility, chest expansion, global well-being, quality of life, and fatigue [2–5]. Research has suggested that AS is associated with increased risk of cardiovascular morbidity [6,7] which would provide an additional indication for AS patients to develop and maintain cardiovascular fitness. Current recommendations for the management of AS include appropriate medication and exercise for all AS patients as core recommendations [8]. In fact, recent recommendations suggest that physiotherapy should be started as soon as AS is diagnosed, at all disease stages and using group physiotherapy and spa exercises [9]. Benefits of home and group-based exercise have been observed [10,11], a the majority of the people with AS do not exercise frequently, with just 18% [12], 23% [13,14], and 34.4% [15] of the patients regularly exercising in different studies. The main barriers were due to fatigue’ with 71% reporting that “exercise tires me” or “I am fatigued by exercise” [14,15]. It can be argued that exercise is undertaken by the less severe patients and is really only feasible for those with low pain and fatigue levels. This means exercise may appear to improve the outcome of the AS but that this is actually reverse causation, in that those with severe pain, fatigue, and disease activity cannot exercise, while those with milder disease can and do exercise.

Here, we set out to investigate this by controlling for disease severity and examining the effect of exercise on function in AS.

## Methods

### Study design

Cohort study.

### Participants

As part of the Medical Research Council/NISCHR Patient Research Cohort Initiative, people with AS were recruited and asked to complete questionnaires about multiple aspects of their disease every 3–6 months. The protocol for this study has previously been published [16] and involves 572 patients recruited through their rheumatologist or general practitioner between 2009 and 2011. These questionnaires captured AS-specific disease activity and function and quality of life.

A separate exercise questionnaire was circulated 1 year into the cohort study. This was sent to a total of 371 people, as 201 patients had an incomplete set of questionnaires concerning other aspects of their disease (primarily due to being recently recruited), had dropped out of the cohort, moved, or died. The exercise questionnaire included instruments related to physical activity and motivation to exercise.

Three months after completion of the exercise questionnaire’ patients were surveyed again in relation to disease activity and function.

### Rating instruments

#### Behavioral Regulation in Exercise Questionnaire

Participants were requested to complete the validated modified Behavioral Regulation in Exercise Questionnaire (BREQ-2) [17] which explores motivation to exercise. The BREQ-2 consists of 19 items which measure the stages of self-determination on a continuum from amotivation (lack of motivation), external regulation (exercising to obtain an external reward), introjected regulation (exercising in order to avoid negative feelings, for example guilt, associated with not exercising), identified regulation (recognizing the benefits of exercise for oneself)’ and intrinsic regulation (choosing freely to exercise). The unidimensional relative autonomy index (RAI) [18], derived from the subscales of BREQ-2, is used here and gives an indication of the degree of self-determination to exercise.

#### International Physical Activity Questionnaire—short form

Physical activity was assessed using the International Physical Activity Questionnaire-ShortForm (IPAQ-SF) [19], a validated instrument comprising of four items that estimates the levels of vigorous activity, moderate activity, walking and time spent sitting during the previous 7 days. The volume of an activity is computed by weighting each type of activity by its energy requirements defined in metabolic equivalent tasks (METs) [19] and multiplying the MET score by the time (in minutes) the activity was performed for. Scores are presented as MET-minutes per week. The IPAQ-SF classifies populations into low (scores of <599), moderate (scores between 600 and 2999)’ or high physical activity level (scores over 3000) groups.

#### EQ5D

The EQ5D is a health status/quality of life questionnaire which comprises of five dimensions: mobility, self-care, usual activities, pain/discomfort’ and anxiety/depression. Each dimension has three levels: no problems, some problems, severe problems. The dimensions are scored as 1, 2, or 3. A score of 11111 indicates no problem on any of the five dimensions, a score of 11223 would indicate no problem with mobility and self-care but some problems with usual activities and moderate pain/discomfort and extreme anxiety and depression. The score is then translated using a country appropriate single summary score. A score of 1 indicates no impairment, a score below 1 indicates lower quality of life/health status, 0 indicates death and negative scores indicate a health state/quality of life worse than death. These scores can be used to calculate quality adjusted life years (QALY).

#### Hospital Anxiety and Depression Scale

The Hospital Anxiety and Depression Scale [20,21] has 14 questions relevant to anxiety (7 statements) and depression (7 statements). Each question has four possible responses which are scored on a scale of 0–3, the maximum score is 21 for depression and 21 for anxiety with values of 11 or more on the relevant subscale indicating probable depression. Depression has been found to be associated with functional limitations in RA [22,23] and AS [24] and psychological factors such as helplessness and depression serve as mediators in the link between disease activity and functional limitations [24].

#### Bath AS-indices

Other measures collected during the study were Bath Ankylosing Spondylitis Disease Activity Index (BASDAI) [25] and the Bath Ankylosing Spondylitis Functional Index (BASFI) [26]. BASDAI is a self-reported, 6-item questionnaire measuring disease activity on a 10-cm visual analog scale, from none (0 mm) to very severe (100 mm). Items included are severity of fatigue, spinal and peripheral joint pain, localized tenderness’ and morning stiffness over the past week. The lower the score the lower the disease activity. The minimum clinically important difference (MCID), which is the minimum level of change of an outcome measure that is considered to be clinically relevant, for BASDAI was 10 mm (scale 0–100) [27]. BASFI is a self-reported, 10-item questionnaire’ evaluating the ability to function and cope with activities of daily living over the past week using a 10-cm VAS scale, from no limitations (0 mm) to very severe functional limitation (100 mm). The lower the score the better the function. The higher the score the more impaired the function. The MCID for BASFI was 7 mm [27].

### Ethical approval

The study was given favorable ethical approval by the London Multi-centre Research Ethics committee [16].

### Statistical analysis

Descriptive statistics were produced for all variables and exploratory analyses examined correlations of factors related to variation in both current function and in future function. The proportions of participants who reported different types of motivation regulation were reported using descriptive statistics and terciles were used to divide the BASDAI into low, moderate, and high groups. Based on the results from the exploratory analysis’ a path analysis using the *sem* command in STATA 12, was performed. All meaningful relationships were first considered and those that were non-significant (*P*>0.05) were eliminated from the final model. Path analysis allows two regression models to be run together therefore’ taking into account correlations on the path to the dependent variable (functional score). The *r*^2^ value was used to assess the proportion of the variation in function which can be accounted for by the model. The likelihood ratio test was used to test model fit.

## Results

The exercise questionnaire was completed by 326 out of 371 participants (88% response rate). The sample was 79% male, mean age 55 (SD ±14), with a mean disease duration of 22 years (SD ±15) from the date of diagnosis and 30 years (SD ±15) from the date of first symptoms. The average BASDAI and BASFI scores were 41 (SD±24) and 47 (SD±29), respectively on a scale of 0–100. Those patients with poor function were older and had higher anxiety and depression scores and lower quality of life scores but there was no difference in gender or medication prescription (Table 1).

Patients with high levels of disease activity (high pain, fatigue, stiffness, BASDAI>60 (scale 0–100)), had a lower average physical activity score compared to those with moderate and mild disease activity (high disease severity—2017 MET-min/wk, moderate disease severity—3347 MET-min/wk and mild disease severity—3255 MET-min/wk respectively, *P* = 0.0028). The cut-offs for low activity are<600, moderate activity 600–2999, high activity>3000 MET-min/wk. Thus, those with moderate and low disease activity do report high levels of physical activity per week.

### Effect of physical activity on function (BASFI) when controlling for disease severity (BASDAI)

BASFI is a measure of functional impairment and so a lower BASFI score will indicate better functional ability and less disability. Higher physical activity is associated with better function in patients with moderate and severe disease activity (Table 2). From Table 2 it can be seen that each unit increase in physical activity category is associated with an improved function score. However, patients with more severe disease activity show more change (improved function) with each increase in physical activity category. For example, a medium level of activity for those with moderate disease activity (BASDAI 35–59) is associated with an improved function (compared to those in the low activity category) of 15.4 on a 1–100 BASFI scale (1.5 cm on the 1–10 cm VAS scale) which is both a statistically significant change and twice the clinically relevant change [27]. However, for those with milder disease (BASDAI less than 35) a high level of activity is needed to see a comparable improvement in function. In this analysis, a high BASDAI indicates high disease activity (increased pain, fatigue) and a high BASFI indicates impaired function. Therefore, a negative coefficient indicates an improvement in function.

### Effect of motivation to exercise on function (BASFI) when controlling for disease severity (BASDAI)

Increasing motivation to exercise is associated with better function for patients with severe disease activity (Table 3). At lower levels of disease activity (milder disease, BASDAI<35) patients need to be highly motivated in order to see significant improvements in function. The effect of depression and anxiety was examined using the HADS [21] score but this did not have a significant effect on function (*P* = 0.97) when adjusted for disease severity.

### Path analysis examining effect of motivation and physical activity on function

#### Current function

**Figure 1:**
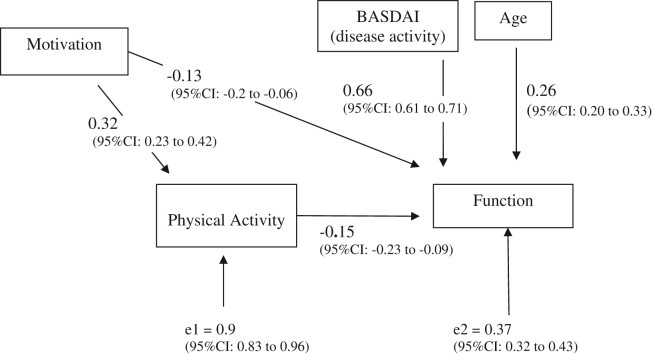
Model of factors associated with current function for individuals with AS (using standardized regression coefficients (*β*)).

We used path analysis to fit two regression models together and examine the effect of motivation and exercise on function (see Fig. 1). We examined the effect of physical activity on current function (see Fig. 1) and found that higher disease activity (pain/fatigue) and aging are the main factors which are associated with loss of function. However, both motivation and physical activity have an effect in increasing function. If motivation increases this has a positive effect on function both directly and indirectly by influencing physical activity levels. Therefore, changing motivation to exercise will have a greater effect than just improving a patient's exercise levels in isolation. A single standard deviation change in motivation is directly associated with an increased function of 0.14 of a standard deviation and indirectly improves function by increasing activity by 0.34 of a standard deviation which will in turn, improve function. The *r*^2^ of this model was 0.63 indicating a significant proportion of the variation in function can be accounted for by the variables in this model. The likelihood ratio test showed good model fit (*P* = 0.7 or no significant difference between model predicted/expected and observed data). The BASDAI (disease activity) did not significantly affect level of physical activity when adjusted for motivation.

#### Future function

**Figure 2:**
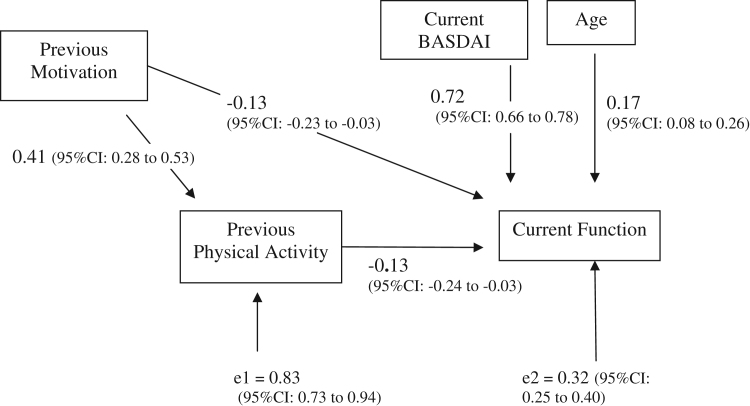
Model of effect of previous motivation and physical activity on function for individuals with AS (using standardized regression coefficients (*β*)).

We examined the effect of physical activity on future function. The current activity was measured in the exercise questionnaire and function was assessed 3 months later (Fig. 2). Motivation and physical activity influences future function in that higher levels of previous activity and higher motivation to exercise in the past lead to lower functional impairment. For example, a 1 standard deviation change in previous motivation leads to a 0.13 standard deviation improvement in current function and a 0.41 standard deviation increase in physical activity levels. A 1 standard deviation change in past physical activity leads to a 0.13 standard deviation improvement in current function. The *r*^2^ of this model was 0.68 indicating a significant proportion of the variation in function can be accounted for using age, current disease activity, previous motivation level’ and previous physical activity level. The likelihood ratio test showed a good fit of the model (*P* = 0.1, showing no significant difference in observed [data] and expected [model predicted]). The BASDAI (disease activity) did not significantly affect level of physical activity when adjusted for motivation.

Table 4 shows the analysis of the important factors in regulating motivation in patients and their influence on exercise and function. This highlights that intrinsic motivation (freely choosing to exercise because it is pleasurable and fun) is associated with the greatest effect on exercise level and benefit in function. Identified regulation (perceiving exercise as fundamentally important and beneficial) was also important, as was introjected regulation (exercising to avoid feeling guilty or ashamed) to a minor degree. External motivation (to please others) was of no benefit.

## Discussion

This study was conducted to explore the effect of exercise and motivation to exercise on the functional ability of people with AS, while controlling for severity, a potential confounding factor of function, exercise, and motivation.

We found that exercise does improve function when controlling for disease activity (pain/fatigue). It is at the higher levels of disease activity (pain/fatigue) that smaller changes in activity will have most improvement in function. In addition, motivation to exercise improves function both indirectly, by increasing exercise levels, and directly. Therefore, interventions aimed at enhancing motivation to exercise comined with provision of access to exercise would improve function 2-fold, compared to providing access to exercise programmes and facilities alone.

Our findings are in line with findings from studies in other forms of arthritis. For example, studies with rheumatoid arthritis (RA) patients have found that lack of motivation and lack of beliefs related to the benefits of exercise are factors strongly associated with inactivity [28,29] and motivation significantly determines physical activity levels, with a more autonomous motivation style predicting higher levels of physical activity [29,30]. Thus, improving self-efficacy is as important in improving physical activity. Intrinsic and identified motivation appear particularly important, so exercise referral or advising physiotherapy are unlikely to result in improved exercise or function. Instead strategies should include working with people to improve intrinsic motivation such as motivational interviewing and goal setting.

People with RA and knee osteoarthritis in the lowest quartile in terms of function, when given physical activity coaching, had the largest mean physical improvement over baseline [31]. Therefore, it is the older, more disabled patients who should be targeted with improving activity as these are the patients who benefit most.

We found people with higher disease activity undertook less physical activity compared to those with moderate to mild disease activity. This finding is similar to that in RA in that the younger, higher educated, lower disease activity, autonomous regulated patients that had higher activity levels [30]. However, our study differs from other studies which have found that people with more pain (and osteoarthritis) were more motivated (though not necessarily more active) [32] and in AS, it has previously been found that people with more disability were found to exercise more frequently than those with less disability [12].

### Limitations of the study and future work

The physical activity measure in this study is self-reported and therefore the levels of physical activity may not always be accurate. Often people over estimate their activity and future studies should use a combination of methods such as a questionnaire combined with pedometer or accelerometer in order to get accurate estimates of activity [33].

In addition, the sample consists of patients who are enthusiastic to complete questionnaires about their AS and may represent an inherently motivated group that is not representative of the general AS population.

Finally, in patients with AS, disease activity correlates weakly with acute phase responses; however, acute phase response may have an effect on fatigue and relatively motivation to exercise. We used the BASDAI which does not include acute phase reactants, but the Ankylosing Spondylitis Disease Activity Score [34] does include acute phase response and it is possible that a study using ASDAS may present slightly different results.

Future work needs to examine how to enhance motivation as wells as provide activity facilities and programmes. Enhancement of motivation can be achieved by a variety of approaches, ranging in complexity and style from the basic educational, to behavioral activity diaries and goal setting (including motivational interviewing), through to more comprehensive cognitive behavioral therapy and mindfulness-based interventions. For example, a unique and tailored health-promotion intervention aimed at increasing physical activity levels in individuals with arthritis has been described and emphasizes motivational interviewing, individualized goal setting, tailored strategies for increasing activity, and monitoring progress and an assessment of barriers and drivers to physical activity [35]. In addition, for people with arthritis potential strategies for increasing exercise participation include incorporating pain management strategies and coping skills into exercise interventions [36]. Mindfulness-basedcognitive therapy (MBCT) and Mindfulness-BasedStress Reduction (MBSR) have been shown to be efficacious for depression in RA [37], pain in osteoarthritis [38], and also fatigue in multiple sclerosis [39]. Additionally, behavioral therapy has already been shown to have benefits for chronic pain and fatigue in arthritic conditions [40], as well as increase adherence to exercise programmes in obesity [41]. Thus, incorporating motivation interventions within exercise programmes could allow people with AS to undertake increased self-management, especially for those with higher disease activity levels, where the observed effects on function were more pronounced. Our study suggests that it is psychological interventions that result in improved intrinsic motivation that may provide the most benefit for patients—mindfulness-based interventions in particular could be of value.

## Conclusions

Both exercise and motivation to exercise have a significant effect on the functional ability of people with AS. Motivation to exercise is also just as important as exercise levels and has a direct effect on function as well as an additional indirect effect through improving activity levels. Exercise is an important factor in maintaining function for people with AS and is particularly important for those with higher disease activity levels.

## Figures and Tables

**Table 1:** Demographic Table by Functional Level

	Good Function (score<50), *n* = 194	Poor Function (score>50), *n* = 167	Difference (95% CI)
Male gender (*n*)	81.5% (155)	76.5% (127)	–
Age, mean (SD)	51.7 (14.5)	58.4 (11.7)	6.7 (3.9–9.4)
Anti TNF use (*n*)	13.9% (27)	18.6% (31)	–
Non-steroidal use (*n*)	86% (167)	79% (132)	–
EQ5D, mean (SD)	0.75 (0.2)	0.41 (0.3)	0.33 (0.28–0.38)
HAD score, mean (SD)	12.9 (5.3)	15.5 (5.9)	2.6 (1.47–3.8)
Disease severity (scale 0–100)	27.7 (18.6)	56.8 (18.5)	29 (25.2–32.9)

Exercise			
Low	12.9% (25)	49.7% (83)	36% (27–45%)
Medium	43% (84)	29.3% (49)	14% (4–23.5%)
High	43.8% (85)	21% (35)	23% (13–31.8%)

**Table 2:** Improvement in Function (BASFI) with Physical Activity Level When Stratified by Disease Severity and Controlled For Age

	Impairment in Function Score
	Slope/Coefficient (95% CI)
*Low disease activity BASDAI<35 (scale 0–100)*	
Physical activity	
Low	1
Medium	−8.9 (−17.9 to 0.02)
High	−14.0 (−23.3 to−4.8)⁎
Age	0.39 (0.16–0.6)⁎

*Moderate disease activity BASDAI 35–59 (scale 0–100)*	
Physical activity	
Low	1
Medium	−15.4 (−24.4 to−6.5)⁎
High	−21.3 (−30.2 to 12.5)⁎
Age	0.63 (0.37–0.89)⁎

*High disease activity BASDAI>60 (scale 0–100)*	
Physical activity	
Low	1
Medium	−8.0 (−16.0 to−0.08)⁎
High	−19.9 (−27.9 to−11.8)⁎
Age	0.46 (0.22–0.7)⁎

⁎ Significant (*P*<0.05), a negative coefficient indicates improvement in function. A positive coefficient indicates poorer function.

**Table 3:** The Effect of Motivation to Exercise on the Functional Ability (BASFI) of AS Patients

	Impairment in Function Score
	Slope/Coefficient (95% CI)
*Low disease activity BASDAI<35 (scale 0–100)*	
Motivation	
None	1
Moderate	4.8 (−5.2 to 15.0)
Motivated	−5.9 (−15.0 to 3.2)
High	−11.22 (−19.7 to−2.7)⁎
Age	0.48 (0.25–0.7)⁎

*Moderate disease activity BASDAI 35–59 (scale 0–100)*	
Motivation	
None	1
Moderate	−6.9 (−18.6 to 4.6)
Motivated	−13.6 (−25.3 to−1.9)⁎
High	−16.9 (−28.4 to−5.3)⁎
Age	0.63 (0.35–0.91)⁎

*High disease activity BASDAI>60 (scale 0–100)*	
Motivation	
None	1
Moderate	−17.5 (−26.4 to−8.5)⁎
Motivated	−21.5 (−33.1 to−9.8)⁎
High	−24.2 (−37.4 to−11.1)⁎
Age	0.65 (0.33–0.97)⁎

⁎ Significant (*P*<0.05), a negative coefficient indicates improvement in function. A positive coefficient indicates poorer function.

**Table 4:** Mode of Motivation Regulation and Exercise and Function Regulation

	Amotivation	External	Introjected	Identified	Intrinsic
Description	“I can't see why I should bother to exercise”	“To please other people”	I feel guilty when I don't exercise”	“Because I value the benefits of exercise”	“Because I think exercise is fun”
Exercise *β* slope	−667 (−1242 to−92)⁎	86.33 (−433 to 606)	274 (−91 to 638)	994 (651.5–1336)⁎	1320 (960–1680)⁎
Function *β* slope	6.6 (2.2–11.1)⁎	−0.1 (−4.1 to 3.9)	−3.9 (−6.7 to−1.1)⁎	−7.3 (−10.1 to−4.7)⁎	−11.7 (−14.3 to−8.9)⁎

⁎ Significant (*P*<0.05).
